# NAFLD in Polycystic Ovary Syndrome: Association with *PNPLA3* and Metabolic Features

**DOI:** 10.3390/biomedicines10112719

**Published:** 2022-10-27

**Authors:** Amanda Medeiros Recuero, Larissa Garcia Gomes, Gustavo Arantes Rosa Maciel, Fernanda de Mello Malta, Ana Paula Moreira Salles, Denise Cerqueira Paranaguá Vezozzo, Edmund Chada Baracat, João Renato Rebello Pinho, Flair José Carrilho, José Tadeu Stefano, Claudia P. Oliveira

**Affiliations:** 1 Laboratório de Gastroenterologia Clínica e Experimental (LIM-07), Division of Clinical Gastroenterology and Hepatology, Department of Gastroenterology, Hospital das Clínicas, School of Medicine, University of Sao Paulo, Sao Paulo 048293, Brazil; 2 Division of Clinical Gastroenterology and Hepatology, Department of Gastroenterology, Hospital das Clínicas, School of Medicine, University of São Paulo, Sao Paulo 048293, Brazil; 3 Division of Endocrinology and Metabolism, School of Medicine, University of Sao Paulo, Sao Paulo 048293, Brazil; 4 Division of Gynecological Clinic, School of Medicine, University of Sao Paulo, Sao Paulo 048293, Brazil; 5 Institute of Tropical Medicine, LIM 07, University of Sao Paulo, Sao Paulo 048293, Brazil

**Keywords:** NAFLD, PCOS, insulin resistance, patatin-like phospholipase domain containing 3

## Abstract

Background: The aim of this study was to determine the frequency of the rs738409 polymorphism in the patatin-like phospholipase domain containing 3 (*PNPLA3*) gene in patients with polycystic ovary syndrome (PCOS) and its impact on nonalcoholic fatty liver disease (NAFLD) risk and severity. We also evaluated other risk factors associated with NAFLD and advanced fibrosis. Methods: This was a cross-sectional study involving 163 patients with PCOS at a tertiary center. Genotyping for the *PNPLA3* polymorphism was undertaken using a TaqMan assay. The degree of fibrosis was defined by transient elastography. Results: The prevalence of NAFLD was 72.4%, and the polymorphism was heterozygous in 41.7% and homozygous in 8% of patients. Homeostasis model assessment of insulin resistance ≥ 2.5 was the main factor associated with the risk of developing NAFLD (OR = 4.313, *p* = 0.022), and its effect was amplified by the polymorphism (OR = 12.198, *p* = 0.017). Age > 32 years also conferred a higher risk for NAFLD. HDL values ≥ 50 mg/dL conferred protection against the outcome. Metabolic syndrome (OR = 13.030, *p* = 0.020) and AST > 32 U/L (OR = 9.039, *p* = 0.009) were independent risk factors for advanced fibrosis. Conclusions: In women with PCOS, metabolic characteristics are more relevant than *PNPLA3* polymorphism regarding the risk for NAFLD and its advanced forms, but these factors can act synergistically, increasing disease risk.

## 1. Introduction

Nonalcoholic fatty liver disease (NAFLD) is currently the most common cause of liver disease in Western populations, with a prevalence as high as 33% in the general population [1]. NAFLD is strongly associated with metabolic syndrome (MS), as well as with its components, such as obesity, dyslipidemia, systemic arterial hypertension (SAH), high blood glucose levels and insulin resistance (IR) [2]. Recent advances have allowed a better understanding of the role of genetic factors in the development and progression of NAFLD. The rs738409 polymorphism in the patatin-like phospholipase domain-containing 3 (*PNPLA3*) gene was identified in a genome-wide association study (GWAS) as the main genetic factor associated with the different forms of NAFLD manifestations [3]. Numerous other subsequent studies have reproduced the relationship between polymorphisms in the *PNPLA3* gene and NAFLD [4], linking the I148M variant not only with the development of the disease but also with a greater predisposition to progression with fibrosis [5].

Polycystic ovary syndrome (PCOS) is the most common endocrine disorder affecting women of reproductive age, with an estimated prevalence of 6 to 15% [6]. Women with PCOS are at an increased risk of developing NAFLD, with a prevalence of 35 to 70% [7], and develop the disease earlier than do other groups, in addition to presenting more advanced forms [7]. Although *PNPLA3* polymorphism is the main genetic risk factor for NAFLD and additionally modulates response to its treatment [8], there have been no studies that have evaluated its role in the development of fatty liver in women with PCOS. The purpose of this study was to evaluate the prevalence and impact of the I148M *PNPLA3* polymorphism on the development of NAFLD and its progression to hepatic fibrosis in women with PCOS and to identify other possible risk factors associated with these outcomes.

## 2. Materials and Methods

### 2.1. Ethical Aspects

The Ethics Committee of the Clinics Hospital of the University of São Paulo Medical School approved this study (opinion number 2.554.658). The study was conducted in accordance with the 2013 Declaration of Helsinki, and signed written consent forms were obtained from all patients.

### 2.2. Study Design and Population

This was a cross-sectional study that evaluated patients with PCOS treated between 2017 and 2019 in the outpatient clinics of the Departments of Gastroenterology, Endocrinology and Gynecology at the Clinics Hospital of the University of São Paulo Medical School, Brazil. The inclusion criteria were as follows: any patient diagnosed with PCOS according to Rotterdam 2003 criteria [9] in whom the presence of 2 of the following presentations were used to confirm the diagnosis: clinical hyperandrogenism, ovulatory dysfunction and polycystic ovary morphology (PCOM) on ultrasound (US), after the exclusion of secondary causes of the respective changes. The characteristics of PCOS were grouped and classified into phenotypes according to previously published studies [10] as follows: phenotype A (hyperandrogenism + ovulatory dysfunction + PCOM), phenotype B (hyperandrogenism + ovulatory dysfunction), phenotype C (hyperandrogenism + PCOM) and phenotype D (ovulatory dysfunction + PCOM).

### 2.3. Exclusion Criteria

The exclusion criteria were as follows: (1) alcohol consumption ≥ 20 g per day; (2) presence of secondary causes of liver disease (viral hepatitis, autoimmune hepatitis, Wilson’s disease, hemochromatosis, celiac disease, cholestatic liver diseases, previous or current use of hepatotoxic drugs, alpha-1-antitrypsin deficiency); (3) patients with human immunodeficiency virus (HIV); (4) pregnancy at the time of recruitment; and (5) refusal to participate in the study.

### 2.4. Diagnosis of NAFLD and Assessment of the Degree of Liver Fibrosis

The presence of hepatic steatosis was evaluated by abdominal US performed by an experienced radiologist with a Toshiba SSA-240A^®^ device (Toshiba; Tokyo, Japan). Tests were conducted to screen for secondary causes of liver disease. All patients diagnosed with NAFLD were subjected to transient elastography (TE) using a Fibroscan^®^ (Echosens, Paris, France) device equipped with M and XL probes and performed by an experienced examiner. The test was performed under fasting for at least 4 h. The XL probe was used in patients with a body mass index (BMI) ≥ 30 kg/m^2^ and skin-capsular distance > 2.5 cm. Data corresponding to the estimate of the degree of liver stiffness (liver stiffness measurement (LSM)), expressed in kilopascals (kPa), were obtained, requiring a minimum of 10 valid measurements, with a success rate > 60% and interquartile range ≤ 30% to define the test as valid. The following cutoff values were used in the staging of the degree of fibrosis: mild fibrosis (F1), LSM 5.8 to 6.9 kPa; clinically significant fibrosis (F2), 7.0 to 8.6 kPa; advanced fibrosis (F3), 8.7 to 11.4 kPa; and cirrhosis (F4), >11.4 kPa [11].

An evaluation of the controlled attenuation parameter (CAP), expressed in decibels per meter (dB/m), was also performed to define the degree of hepatic steatosis. The correspondence of the CAP values obtained with the degree of steatosis was determined using the following cutoff values: grade 1 (G1), 231–267 dB/m; grade 2 (G2), 268–300 dB/m; and grade 3 (G3), >300 dB/m [12].

### 2.5. Extraction of Genomic DNA

DNA was extracted from whole blood samples using a QIAamp DNA^®^ Blood mini kit (Qiagen^®^, Hilden, Germany), following the manufacturer’s instructions. The *PNPLA3* gene polymorphism (rs738409) genotype was assessed in all samples by a team who were not aware of the patient’s clinical condition. The extracted DNA was used in subsequent real-time polymerase chain reactions, which were performed using StepOne Plus equipment (Applied Biosystems, Foster City, CA, USA). The reaction systems included TaqMan Genotyping Master Mix, and TaqMan SNP Genotyping Assay reagents (Applied Biosystems, Foster City, CA, USA) were used. Quality controls were performed to verify the reproducibility of the results.

### 2.6. Variables Evaluated in the Study

Demographic and anthropometric variables ((age, height, waist circumference (WC), hip circumference (HC), waist-hip ratio (WHR) and BMI) were obtained at the time of the medical consultation. Patients were classified as follows: underweight (<18.5 kg/m^2^), normal weight (18.5–24.9 kg/m^2^), overweight (25–29.9 kg/m^2^), class I obesity (30–34.9 kg/m^2^), class II obesity (35–39.9 kg/m^2^) and class III obesity (>40 kg/m^2^) [13]. A previous history of smoking was also studied. Samples for the biochemical and hormonal serum tests were collected after 12 h of fasting. The patients underwent an oral glucose tolerance test (75 g of anhydrous glucose) with measurement of basal glucose and insulin and measurement at 120 min after the administration of glucose.

The fibrosis-4 (FIB-4) index was calculated using the formula FIB-4 = age (years) × aspartate aminotransferase (AST) (IU/L)/[platelet count (10^9^/L) × alanine aminotransferase (ALT) (UI/L)^1/2^] [14]. The NAFLD fibrosis score (NAFLD-FS) was calculated using the following formula: NAFLD-FS = −1.675 + 0.037-age (years) + 0.094 × BMI (kg/m^2^) + 1.13 × impaired fasting glucose/diabetes (yes = 1, no = 0) + 0.99 × AST/ALT ratio—0.013 × platelet count (× 10^9^/L)—0.66 × albumin (g/dL) [15].

The presence of the following comorbidities was evaluated: diabetes (fasting glucose ≥ 126 mg/dL and/or blood glucose 120 min ≥ 200 mg/dL and/or glycated hemoglobin (HbA1c) ≥ 6.5%), prediabetes (fasting glucose ≥ 100 and <126 mg/dL and/or blood glucose 120 min ≥ 140 and <200 mg/dL and/or HbA1c ≥ 5.7 and <6.5%) [16] and SAH (systolic pressure ≥ 130 mmHg or diastolic pressure ≥ 80 mmHg) [17]. Insulin resistance was estimated using homeostasis model assessment of insulin resistance (HOMA-IR), calculated using the following formula: [fasting insulin (mU/L) × fasting glucose (mmol/L)]/22.5 [18]. A value ≥ 2.5 was used as a cutoff point to define IR [19]. Metabolic syndrome was defined according to the recommendations of the Adult Treatment Panel III Report [20]: fasting glucose ≥ 100 mg/dL, triglycerides (TGs) ≥ 150 mg/dL and HDL < 50 mg/dL; systolic pressure ≥ 130 mmHg or diastolic pressure ≥ 85 mmHg; and abdominal obesity (WC ≥ 88 cm).

### 2.7. Statistical Analysis

The descriptive tables of the qualitative variables included the absolute and relative frequencies, 95% confidence intervals (95% CIs) and descriptive levels, and the quantitative variables included measures of central tendency and dispersion. To study the distribution of the qualitative variables, the chi-square test or Fisher’s exact test was used when appropriate. The Mann–Whitney test was used to compare the distribution of the quantitative variables. Hardy–Weinberg equilibrium was evaluated for the sample in question. Heterozygosity and polymorphism information content (PIC) were calculated for the sample and among the interest groups. Different binary logistic regression models were proposed to evaluate the joint impact of the factors of interest in relation to the presence of hepatic steatosis and advanced fibrosis. The hierarchical strategy was used as a variable selection method. The Hosmer–Lemeshow test was used to assess the quality-of-fit model. An ROC curve was used to verify the predictive ability of each model.

All tests considered a bidirectional α of 0.05 and the 95% CI and were performed with computational support from R software (https://www.r-project.org/, accessed on 25 April 2020), IBM SPSS Statistics for Windows, version 25 (IBM Corp., Armonk, NY, USA) and Excel 2016^®^ (Microsoft Office, Redmond, WA, USA). 

## 3. Results

A total of 172 patients were recruited, of whom 7 were excluded due to refusal to perform tests, 1 was excluded due to hepatic steatosis associated with alpha-1-antitrypsin deficiency and 1 was excluded due to evidence of drug-induced hepatitis after liver biopsy. The final study population was 163 women with PCOS.

The mean age of the study population was 32.5 ± 8.1 years, and the population included patients between the ages of 16 and 56 years. The prevalence of NAFLD was 72.4%. The heterozygous (CG) *PNPLA3* gene polymorphism occurred in 41.7% of the study population, and the homozygous (GG) *PNPLA3* gene polymorphism occurred in 8.0%. In the group of women with NAFLD, the prevalence of the CG/GG polymorphism was 53.4%, and in the group without NAFLD, the prevalence was 40.0%, with no statistically significant difference between the groups. The *PNPLA3* polymorphism was in Hardy–Weinberg equilibrium between the groups. The overall heterozygosity of *PNPLA3* in the sample was 0.486, with a polymorphism information content (PIC) of 0.368. The heterozygosity was 0.493, with a PIC of 0.371, among women with NAFLD, and 0.458, with a PIC of 0.353, among women without NAFLD. Among women with advanced fibrosis (≥F3), heterozygosity was 0.408, with a PIC of 0.325, similar to the group without advanced fibrosis (≤F2), whose heterozygosity was 0.481, with a PIC of 0.365.

Patients with evidence of hepatic steatosis by controlled attenuation parameter (CAP) were distributed as follows: 22% with G1 steatosis, 19.5% with G2 steatosis and 51.7% with G3 steatosis. Regarding liver stiffness measurement (LSM), 22.0% of the patients had values corresponding to grade 1 fibrosis, 11.9% to grade 2 fibrosis, 10.2% to grade 3 fibrosis and 1.7% to liver cirrhosis. Regarding the characteristics of PCOS in the studied population, phenotype A was the most common, present in 73.8% of the women. The main characteristics of the study population are provided in Table 1.

All variables studied were stratified according to the presence of the *PNPLA3* polymorphism. There were no statistically significant differences in the prevalence of NAFLD, TE values or metabolic profiles between the women with the presence of the G allele and the others. The mean age of the women who carried the polymorphism was 3 years older than that of the other women. Total testosterone (*p* = 0.041) and free testosterone (*p* = 0.031) values were lower in individuals with the polymorphism, as can be seen in Supplementary Table S1.

In the univariate analysis, PCOM on US, changes in the glycemic profile, MS, HOMA-IR ≥ 2.5, systemic arterial hypertension, use of metformin and different degrees of obesity were associated with the presence of NAFLD. In addition, the following were higher in women with hepatic steatosis: BMI, WC, WHR, total cholesterol, triglycerides, LDL, insulin, fasting glucose, HOMA-IR, HbA1c, AST, ALT, gamma glutamyl transferase (GGT) and ferritin. Importantly, only 21.6% (*n* = 26) of patients with NAFLD had high transaminase levels (reference values: 10–36 U/L), and 35.8% (*n* = 43) had altered GGT levels (reference values: 7–32 U/L). The mean age of the women with steatosis was 5 years older than that of the women without steatosis. Only HDL levels were significantly lower among individuals with steatosis. There was no association between the different PCOS phenotypes and the presence of NAFLD (Supplementary Table S2).

**Figure 1:**
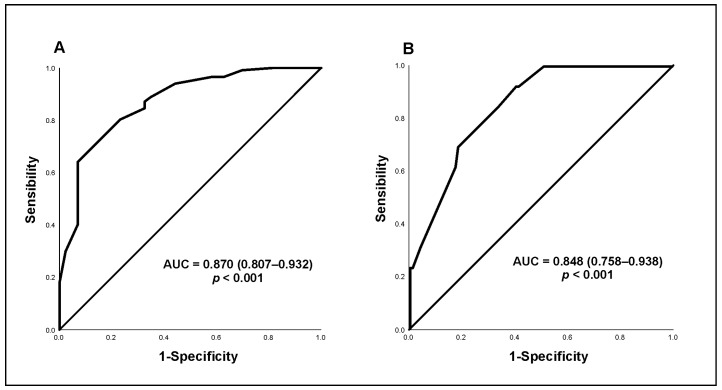
Receiver operating characteristic (ROC) curve for clinical prediction model of NAFLD (**A**) and advanced fibrosis (**B**).

In the logistic regression model adjusted to evaluate the combined effect of the different factors on predicting NAFLD (Table 2), polymorphisms in the *PNPLA3* gene did not present an isolated association with NAFLD. Age greater than 32 years (OR = 3.833, *p* = 0.007) and HOMA-IR ≥ 2.5 (OR = 4.313, *p* = 0.022) were associated with a higher frequency of NAFLD. The interaction of the polymorphism with HOMA-IR ≥ 2.5 resulted in an OR = 12.198 (*p* = 0.017). HDL ≥ 50 mg/dL (OR = 0.237, *p* = 0.004) was associated with the absence of NAFLD. The proposed model showed good accuracy, with an AUC of 0.870 (95% CI: 0.807–0.932; *p* < 0.001) (Figure 1A).

In the analysis of factors associated with the presence of advanced fibrosis (≥F3) on TE, the use of metformin and MS were more frequent in this group. Higher fibrosis-4 score, AST, ALT, WC and WHR values were also observed in women with evidence of advanced fibrosis, with a mean age 6 years older than those with NAFLD without advanced fibrosis (Supplementary Table S3). In the multiple logistic regression model proposed to evaluate the effects of different factors on the occurrence of advanced fibrosis (Table 3), the presence of MS (OR = 13.050, *p* = 0.020) and AST > 32 U/I (OR = 9.039, *p* = 0.009) were significantly associated with the outcome, regardless of age or *PNPLA3* polymorphism. The model in question showed good accuracy, with an AUC of 0.848 (95% CI: 0.758–0.938; *p* < 0.001) (Figure 1B).

## 4. Discussion

In this study, which evaluated 163 women with PCOS, we demonstrated that the *PNPLA3* gene polymorphism had no independent association with NAFLD or its advanced forms or with endocrine and metabolic changes in this population. However, metabolic characteristics seem to play a central role in the risk for NAFLD. IR was the main risk factor for NAFLD in these women, and a synergistic interaction between IR and the polymorphism contributed to an even higher risk of developing the disease. 

In the studied population, the frequencies of the *PNPLA3* CC, CG and GG gene genotypes were 50.3%, 41.7% and 8.0%, respectively. The genotypic distribution was very similar to that observed in a healthy Brazilian population in a recently published study [21]. We also observed a high prevalence of NAFLD in women with PCOS, which affected 72.4% of them. This prevalence is higher than that observed in women of childbearing age without PCOS, in agreement with previous studies [22].

In our population, the prevalence of altered HOMA-IR levels was 75%, and its presence increased the odds of women with PCOS developing NAFLD four-fold. IR is considered a central pathogenic mechanism in the development and progression of NAFLD [23], increasing the supply of hepatic free fatty acids generated by increased lipolysis in adipocytes, stimulating anabolic processes through hyperinsulinemia and favoring the intrahepatic accumulation of lipids [24]. In addition, IR also affects the liver’s ability to suppress glucose production in response to insulin, keeping glycogenolysis active, promoting gluconeogenesis and stimulating de novo lipogenesis [23].

Despite not being correlated with NAFLD as an isolated factor, the G allele in combination with high HOMA-IR levels has a deleterious synergistic effect, generating an even greater risk of NAFLD than that conferred through the isolated effect of HOMA-IR levels. One of the possible explanations for this interaction is that the expression of the *PNPLA3* gene is stimulated by sterol regulatory element binding protein-1c (SREBP-1), an insulin-regulated transcription factor. In the variant form of the protein, the highest amount of adiponutrin (ADPN), stimulated directly by SREBP-1 and indirectly by high insulin levels, accumulates on the surface of lipid droplets by avoiding ubiquitination, thus favoring the accumulation of intrahepatic lipids [25]. 

There were no associations between ADPN variants and the various metabolic changes presented by the women. Similarly, in the same GWAS responsible for the first description of the association of the rs738409 polymorphism of *PNPLA3* with NAFLD, there was no correlation between the polymorphism and metabolic traits [3]. Our results showed lower total and free testosterone levels in women with the rs738409 *PNPLA3* gene polymorphism compared to those in the other women. Despite this finding, there was no significant difference in the distribution of PCOS phenotypes or in the prevalence of hyperandrogenism between the groups.

Although the association between the hyperandrogenic phenotypes of PCOS and NAFLD has already been demonstrated [26], this finding was not demonstrated in our study. Previous research demonstrates a distinct fatty acid profile for the hyperandrogenic and nonhyperandrogenic phenotypes of PCOS, with intensified metabolic pathways of palmitic acid in PCOS patients with normal androgen levels [27], for example. Elevated levels of palmitic acid synthesis have been previously demonstrated in patients with NAFLD [28]. How differences in fatty acid profiles in PCOS phenotypes may have influenced the findings of this research needs to be better elucidated in further studies.

In our study, HDL levels ≥ 50 mg/dL were a protective factor against the development of NAFLD. A low HDL level is a metabolic and cardiovascular risk factor, and the association of low HDL levels with NAFLD has been previously demonstrated [29,30]. Nevertheless, with regard to the NAFLD prediction model in women with PCOS, age over 32 years increased the odds of presenting the disease by almost four-fold. The early development of NAFLD is already well documented in women with PCOS, with advanced stages of fibrosis in women younger than 40 years old [31,32]. A retrospective study that analyzed 602 patients diagnosed with PCOS showed that younger women had higher rates of ovarian dysfunction and hyperandrogenism and that metabolic disorders predominated in women older than 30 years; furthermore, age was also a predictor of insulin levels, with higher IR in the older age group [33]. It is likely that the association of age with a higher risk of NAFLD reflects the strong association with IR and a progressive increase in IR with age.

Women with evidence of fibrosis ≥ F3 were, on average, 6 years older than those with fibrosis ≤ F2. However, they were still younger than 40 years, the age at which there is a higher risk of advanced NAFLD in the general population [34]. The association of the use of metformin with fibrosis levels ≥ F3 is most likely related to the criteria that indicate the use of this medication in this group of women, especially for the treatment of women with PCOS with BMI ≥ 25 and/or metabolic risk factors [35], a group at higher risk for advanced forms of NAFLD.

After evaluating the factors associated with the presence of advanced fibrosis using a multiple logistic regression model adjusted for age, only the presence of MS and AST > 32 U/L maintained significant associations. The main hypothesis regarding the relationship between MS and advanced forms of NAFLD is that IR affects liver lipogenesis and induces high levels of inflammatory mediators, such as interleukin 6, TNFα and c-Jun N-terminal Kinase, leading to liver necroinflammation. Kanwar et al. demonstrated that in patients with MS and NAFLD without diabetes, there is a higher prevalence of IR and greater severity of hepatic steatosis and portal inflammation than in patients without MS [36].

Similar to our findings, those from a study that evaluated 161 patients with NAFLD by means of TE indicated an association of higher levels of AST with advanced fibrosis [37]. The findings of our study, in accordance with the current literature, reinforce the need for liver assessment in patients with PCOS and MS or elevated levels of AST to stratify the risk of NAFLD progression to NASH with advanced forms of fibrosis. Preferably, this assessment should be conducted using noninvasive tools, such as hepatic elastography, given the poor correlation of clinical and laboratory scores with the degree of fibrosis in this population.

Since the presence and severity of liver fibrosis are the main determinants of NAFLD outcomes, it is essential to evaluate this parameter to elucidate the factors associated with poor patient prognosis. Our study used TE, with measurements of the CAP and LSM to evaluate the degree of steatosis and fibrosis, respectively, as an alternative to liver biopsy. In addition to the limitations of liver biopsy, it is ethically questionable to subject women without evidence of liver disease or without clinical indication to this procedure, adding unnecessary risks. Additionally, TE has good discriminatory power for the degree of fibrosis, with an AUC of 0.83 and a negative predictive value of 90% for the categorization of advanced and non-advanced fibrosis [38]. Our population showed a high prevalence of obesity, and the effects of BMI on the adequate measurement of LSM are documented in the literature, especially with regard to obtaining reliable LSMs with the use of the M probe in patients with BMI ≥ 30 kg/m^2^ [39]. However, once the correct probe is used based on BMI and skin-capsular distance, the diagnostic accuracy increases [40]. 

Some limitations of our study are noteworthy. Recruitment in a tertiary center may have contributed to a worse metabolic profile, increasing the prevalence and severity of NAFLD and making the extrapolation of our results to the general population of women with PCOS questionable. An additional limitation was the collection of medical records regarding the phenotypic manifestations of PCOS at diagnosis, not only clinical but also laboratory findings, because a large part of the evaluated population had an established diagnosis of PCOS.

One of the strengths of our study is the fact that we evaluated, in women with PCOS, not only the role of the rs738409 *PNPLA3* polymorphism in the risk of NAFLD and its progressive forms but also its interaction with other metabolic and endocrine factors. Despite the high prevalence of NAFLD in women with PCOS and its potential risk of progression, the indication for screening in these patients is still controversial [41], and many specialists responsible for the care of these patients are unaware of their association [42]. Considering that NAFLD results from a complex interaction between genetic, environmental and metabolic factors, a better understanding of the risk factors associated with this disease and its progression is essential for the proper screening of these patients and for improving their follow-up. In addition, to the best of our knowledge, this study investigated the largest sample of women with PCOS and NAFLD evaluated by means of TE, providing additional data on the risk factors related to the presence of advanced fibrosis, which are still scarce in the literature. It is necessary to take a careful look at women with PCOS and MS due to the increased risk of advanced forms of NAFLD, with fibrosis at early ages, making risk stratification essential in this group.

In summary, we demonstrated that in women with PCOS, IR is the main risk factor for the development of NAFLD and that when interacting with the rs738409 *PNPLA3* polymorphism, the effect is synergistic, contributing to an even higher risk of developing the disease. In contrast to what has been demonstrated in other groups, our results indicate that the development of NAFLD in women with PCOS has a greater association with metabolic changes than with genetic risk. MS and AST > 32 U/L were the main risk factors associated with advanced fibrosis in this population, suggesting the need for additional liver assessments for women with PCOS who have such characteristics for NAFLD risk stratification.

## Figures and Tables

**Table 1:** Demographic, clinical and biochemical characteristics of the population studied (*n* = 163).

	% (*n*) or Mean ± SD
Age, y	32.5 ± 8.1
NAFLD	72.4% (118)
*PNPLA3* polymorphism	
CC	50.3% (82)
CG	41.7% (68)
GG	8.0% (13)
CAP, dB/m *	
<231	6.8% (8)
231–267	22.0% (26)
268–300	19.5% (23)
>300	51.7% (61)
LSM, kPa *	
<5.8	54.2% (64)
5.8–6.9	22.0% (26)
7.0–8.6	11.9% (14)
8.7–11.4	10.2% (12)
>11.4	1.7% (2)
AST, U/L	20.0 ± 9.0
ALT, U/L	25.0 ± 16.0
GGT, U/L	30.0 ± 22.0
Ferritin, ng/mL	112.6 ± 103.4
PCOM	82.4% (131)
HA	95.7% (156)
Irregular menstrual cycles	95.7% (156)
PCOS phenotype	
A	73.8% (118)
B	16.9% (27)
C	5.0% (8)
D	4.4% (7)
Total testosterone, ng/dL	59.1 ± 37.1
Free testosterone, ng/dL	36.0 ± 24.0
Androstenedione, ng/dL	2.4 ± 4.7
DHEA-S, ng/mL	2130.0 ± 1376.0
Glycemic profile	
Normal	19.8% (32)
IR without diabetes or prediabetes	50% (81)
Prediabetes	17.3% (28)
Diabetes	13% (21)
Fasting insulin, µU/mL	27.2 ± 27.5
Fasting glucose, mg/dL	94.0 ± 35.0
HOMA-IR	6.6 ± 6.9
HOMA-IR ≥ 2.5	75% (120)
Metformin use	33.7% (55)
Metabolic syndrome	42.6% (69)
SAH	18.4% (30)
Total cholesterol, mg/dL	182.0 ± 36.0
LDL, mg/dL	107.0 ± 30.0
HDL, mg/dL	49.0 ± 12.0
Triglycerides, mg/dL	151.0 ± 98.0
Smoking status	
Current smoker	5.5% (9)
Former smoker	4.9% (8)
Weight classification by BMI	
Normal	12.3% (20)
Overweight	22.7% (37)
Obesity class I	26.4% (43)
Obesity class II	25.1% (41)
Obesity class III	13.5% (22)
Waist circumference, cm	99.7 ± 17.0
WHR	0.9 ± 0.1

* Transient hepatic elastography was not performed in 45 patients due to the absence of hepatic steatosis on ultrasound. SD: standard deviation; NAFLD: nonalcoholic fatty liver disease; CAP: controlled attenuation parameter; LSM: liver stiffness measurement; AST: aspartate aminotransferase; ALT: alanine aminotransferase; GGT: gamma glutamyl transferase; PCOM: polycystic ovary morphology; HA: hyperandrogenism and/or hyperandrogenemia; PCOS: polycystic ovary syndrome; DHEA-S: dehydroepiandrosterone sulfate; IR: insulin resistance; HOMA-IR: Homeostasis Model Assessment of Insulin Resistance; SAH: systemic arterial hypertension; LDL: low-density lipoprotein; HDL: high-density lipoprotein; BMI: body mass index; WHR: waist–hip ratio.

**Table 2:** Beta coefficients (B), standard errors, *p*-values, odds ratios and confidence intervals (95% CI) for NAFLD prediction.

	B	Standard Error	*p*-Value	Odds Ratio	95% CI
Age > 32 years	1.344	0.495	0.007 *	3.833	1.454–10.106
Polymorphism CG/GG	−0.564	0.782	0.471	0.569	0.123–2.632
HDL ≥ 50 mg/dL	−1.440	0.476	0.003 *	0.237	0.093–0.603
HOMA-IR ≥ 2.5	1.462	0.639	0.022 *	4.313	1.234–15.075
Polymorphism CG/GG * HOMA-IR	2.501	1.049	0.017 *	12.198	1.562–95.243

* *p*-value < 0.05. NAFLD: nonalcoholic fatty liver disease; HDL: high-density lipoprotein; HOMA-IR: Homeostasis Model Assessment of Insulin Resistance.

**Table 3:** Age-adjusted beta coefficients (B), standard errors, *p*-values, odds ratios and confidence intervals (95% CI) for advanced fibrosis prediction.

	B	Standard Error	*p*-Value	Odds Ratio	95% CI
Polymorphism CG/GG	0.947	0.768	0.205	2.640	0.588–11.947
Metabolic syndrome	2.603	1.117	0.020 *	13.050	1.511–120.625
AST > 32 U/L	2.202	0.848	0.009 *	9.039	1.715–47.640

* *p-*value < 0.05. AST: aspartate aminotransferase.

## Data Availability

The datasets generated during and/or analyzed during the current study are available from the corresponding author on reasonable request.

## Supplementary Materials

The following supporting information can be downloaded at: https://www.mdpi.com/article/10.3390/biomedicines10112719/s1. Table S1: Clinical and biochemical characteristics of patients according to PNPLA3 gene polymorphism; Table S2: Clinical and biochemical characteristics of patients according to the presence of NAFLD; Table S3: Characteristics of NAFLD patients according to the presence of advanced fibrosis on TE.
