# Supplementary material for: NAFLD in Polycystic Ovary Syndrome: Association with PNPLA3 and Metabolic Features
Source: Biomedicines. 2022 Oct 27;10(11):2719. doi: 10.3390/biomedicines10112719 (PMC9687705; doi:10.3390/biomedicines10112719)
Supplement: Supplementary file 1 [file biomedicines-10-02719-s001.zip › biomedicines-1956558-supplementary.pdf]

Supplementary Table S1 - Clinical and biochemical characteristics of patients according to PNPLA3 gene polymorphism.

	PNPLA3		p value
	CC (n = 82)<br>%(n) or Mean±SD	CG/GG (n = 81)<br>%(n) or Mean±SD	
Age, y	31.0 ± 8.0	34.0 ± 8.0	0.044
NAFLD	67.1% (55)	77.8% (63)	0.161
CAP, dB/ m*			0.176
< 231	3.6% (2)	9.5% (6)	
231-267	21.8% (12)	22.2% (14)	
268-300	27.3% (15)	12.7% (8)	
> 300	47.3% (26)	55.6% (35)	
LSM, kPa*			0.684
< 5.8	56.4% (31)	52.4% (33)	
5.8-6.9	23.6% (13)	20.6% (13)	
7.0-8.6	12.7% (7)	11.1% (7)	
8.7-11.4	7.4% (4)	12.7% (8)	
> 11.4	-	3.2% (2)	
AST, U/L	18.0 ± 5.0	22.0 ± 11.0	0.056
ALT, U/L	21.0 ± 10.0	28.0 ± 20.0	0.052
GGT, U/L	29.0 ± 21.0	31.0 ± 23.0	0.371
Alkaline phosphatase, U/L	71.0 ± 20.0	73.0 ± 24.0	0.947
Ferritin, ng/mL	110.1 ± 106.9	114.9 ± 100.6	0.492
PCOS phenotype			0.843
A	76.5% (62)	70.9% (56)	
B	14.8% (12)	19.0% (15)	
C	4.9% (4)	5.1% (4)	
D	3.7% (3)	5.1% (4)	
Total testosterone, ng/dL	63.0 ± 33.1	55.0 ± 40.7	0.021
Free testosterone, ng/dL	40.0 ± 25.0	32.0 ± 22.0	0.032
Androstenedione, ng/dL	2.7 ± 6.3	2.0 ± 1.6	0.619
DHEA-S, ng/mL	2189.0 ± 1305.0	2067.0 ±1453.0	0.386
Glycemic profile			0.102
Normal	19.5% (16)	20.0% (16)	
IR without diabetes or prediabetes	58.5% (48)	41.3% (33)	
Prediabetes	9.8% (8)	16.3% (13)	
Diabetes	12.2% (10)	22.5% (18)	
Fasting insulin, µU/mL	25.3 ± 20.0	29.1 ± 28.4	0.686
Fasting glucose, mg/dL	91.0 ± 16.0	97.0 ± 46.0	0.596
HOMA-IR ≥ 2.5	77.8% (63)	72.2% (57)	0.467
Metabolic syndrome	43.2% (35)	42.0% (34)	1.000
SAH	14.6% (12)	22.2% (18)	0.231
HDL, mg/dL	47.0 ± 13.0	50.0 ± 12.0	0.167
Triglycerides, mg/dL	162.0 ± 114.0	140.0 ± 76.0	0.530
Weight classification by BMI			0.997
Normal	12.2% (10)	12.3% (10)	
Overweight	22.0% (18)	23.5% (19)	
Obesity class I	26.8% (22)	25.9% (21)	
Obesity class II	24.4% (20)	25.9% (21)	
Obesity class III	14.6% (12)	12.3% (10)	

*Transient hepatic elastography was not performed in 45 patients due to the absence of hepatic steatosis on ultrasound.

SD: standard deviation; NAFLD: nonalcoholic fatty liver disease; CAP: controlled attenuation parameter; LSM: liver stiffness measurement; AST: aspartate aminotransferase; ALT: alanine aminotransferase; GGT: gamma glutamyl transferase; PCOS: polycystic ovary syndrome; DHEA-S: dehydroepiandrosterone sulfate; IR. insulin resistance; HOMA-IR. *Homeostasis Model*

Assessment of Insulin Resistance; SAH. systemic arterial hypertension; HDL: high-density lipoprotein; BMI: body mass index

Supplementary Table S2 - Clinical and biochemical characteristics of patients according to the presence of NAFLD

	No NAFLD (n = 45) %(n) or Mean±SD	NAFLD (n = 118) %(n) or Mean±SD	p value
Age, y	29.0 ± 8.0	34.0 ± 8.0	0.002*
PNPLA3 polymorphism			
CC	60.0% (27)	46.6% (55)	0.315
CG	35.6% (16)	44.1% (52)	
GG	4.4% (2)	9.3% (11)	
AST, U/L	17.0 ± 4.0	21.0 ± 10.0	0.026*
ALT, U/L	17.0 ± 7.0	27.0 ± 18.0	< 0.001*
GGT, U/L	21.0 ± 17.0	33.0 ± 22.0	< 0.001*
Ferritin, ng/mL	82.0 ± 90.6	123.6 ± 105.9	0.009*
PCOM	93.2% (41)	78.3% (90)	0.035*
HA	93.3% (42)	96.6% (114)	0.396
Irregular menstrual cycles	97.8% (44)	94.9% (112)	0.675
PCOS phenotype			
A	84.1% (37)	69.8% (81)	0.079
B	6.8% (3)	20.7% (24)	
C	2.3% (1)	6.0% (7)	
D	6.8% (3)	3.4% (4)	
Total testosterone, ng/dL	56.0 ± 28.3	60.2 ± 40.1	0.755
Free testosterone, ng/dL	31.0 ± 24.0	37.0 ± 23.0	0.086
Androstenedione, ng/dL	3.2 ± 8.6	2.1 ± 1.5	0.507
DHEA-S, ng/mL	2369.0 ± 1275.0	2039.0 ± 1407.0	0.052
Glycemic profile			< 0.001*
Normal	50.0% (22)	8.5% (10)	
IR without diabetes or prediabetes	43.2% (19)	52.5% (62)	
Prediabetes	2.3% (1)	17.0% (20)	
Diabetes	4.5% (2)	22.0% (26)	
Fasting insulin, µU/mL	16.6 ± 13.9	31.0 ± 26.4	<0.001*
Fasting glucose, mg/dL	85.0 ± 11.0	97.0 ± 40.0	0.009*
HOMA-IR	3.6 ± 3.3	7.7 ± 7.6	<0.001*
HOMA-IR ≥ 2.5	41.9% (18)	87.2% (102)	<0.001*
Metformin use	13.3% (6)	41.5% (49)	0.001*
Metabolic syndrome	13.3% (6)	53.8% (63)	<0.001*
SAH	2.2% (1)	24.6% (29)	<0.001*
Total cholesterol, mg/dL	173.0 ± 28.0	185.0 ± 38.0	0.049*
LDL, mg/dL	98.0 ± 26.0	111.0 ± 31.0	0.017*
HDL, mg/dL	56.0 ± 12.0	46.0 ± 11.0	<0.001*
Triglycerides, mg/dL	98.0 ± 52.0	171.0 ± 104.0	<0.001*
Smoking status			0.567
Current smoker	2.2% (1)	6.8% (8)	
Former smoker	4.4% (2)	5.1% (6)	
Weight classification by BMI			
Normal	35.6% (16)	3.4% (4)	<0.001*
Overweight	26.7% (12)	21.2% (25)	
Obesity class I	15.6% (7)	30.5% (36)	
Obesity class II	15.6% (7)	28.8% (34)	
Obesity class III	6.7% (3)	16.1% (19)	
Waist circumference, cm	87.8 ± 16.9	104.3 ± 14.8	<0.001*
WHR	0.8 ± 0.1	0.9 ±0.1	<0.001*

*p value < 0.05

NAFLD: nonalcoholic fatty liver disease; SD: standard deviation; AST: aspartate aminotransferase; ALT: alanine aminotransferase; GGT: gamma glutamyl transferase; PCOM: polycystic ovary morphology; HA: hyperandrogenism and/or hyperandrogenemia; PCOS:

polycystic ovary syndrome; DHEA-S: dehydroepiandrosterone sulfate; IR. insulin resistance; HOMA-IR. *Homeostasis Model Assessment of Insulin Resistance*; SAH. systemic arterial hypertension; LDL: low-density lipoprotein; HDL: high-density lipoprotein; BMI: body mass index; WHR: waist-hip ratio

Supplementary Table S3 - Characteristics of NAFLD patients according to the presence of advanced fibrosis on TE

	Fibrosis (TE)		p value
	≤ F2 (n = 104) %(n) or Mean±SD	F3/F4 (n = 14) %(n) or Mean±SD	
Age, y	33.0 ± 8.0	39.0 ± 8.0	0.017*
PNPLA3 polymorphism			0.099
CC	49.0% (51)	28.6% (4)	
CG	40.4% (42)	71.4% (10)	
GG	10.6% (11)	-	
CAP, dB/ m			0.939
< 231	6.7% (7)	7.1% (1)	
231-267	23.1% (24)	14.3% (2)	
268-300	19.2% (20)	21.4% (3)	
> 300	51.0% (53)	57.1% (8)	
NAFLD-FS	0.5 ± 0.2	0.7 ± 0.3	0.031*
FIB-4	0.4 ± 0.2	0.5 ± 0.1	0.154
Total bilirubin, mg/dL	0.4 ± 0.2	0.5 ± 0.1	0.154
AST, U/L	20.0 ± 9.0	30.0 ± 14.0	0.005*
ALT, U/L	26.0 ± 16.0	39.0 ± 23.0	0.028*
GGT, U/L	34.0 ± 23.0	27.0 ± 13.0	0.386
Alkaline phosphatase, U/L	72.0 ± 22.0	80.0 ± 23.0	0.109
Platelets, x 10^9 /L	290.0 ± 56.0	298.0 ± 82.0	0.947
Ferritin, ng/mL	124.0 ± 107.3	120.4 ± 97.5	0.951
HA	97.1% (101)	92.9% (13)	0.401
PCOS phenotype			0.493
A	70.9% (73)	61.5% (8)	
B	20.4% (21)	23.1% (3)	
C	5.8% (6)	7.7% (1)	
D	2.9% (3)	7.7% (1)	
Total testosterone, ng/dL	61.2 ± 40.8	53.4 ± 34.8	0.376
Free testosterone, ng/dL	38.0 ± 24.0	31.0 ± 18.0	0.289
Androstenedione, ng/dL	2.0 ± 1.3	2.5 ± 2.2	0.539
DHEA-S, ng/mL	2124.0 ± 1438.0	1455.0 ± 1024.0	0.050
Glycemic profile			0.414
Normal	8.7% (9)	7.1% (1)	
IR without diabetes or prediabetes	54.8% (57)	35.7% (5)	
Prediabetes	15.4% (16)	28.6% (4)	
Diabetes	21.2% (22)	28.6% (4)	
Fasting insulin, μU/mL	31.2 ± 27.6	30.1 ± 14.9	0.460
Fasting glucose, mg/dL	95.0 ± 36.0	115.0 ± 61.0	0.146
HOMA-IR	7.4 ± 7.5	9.5 ± 8.3	0.279
HOMA-IR ≥ 2.5	87.4% (90)	85.7% (12)	1.000
Metformin use	37.5% (39)	71.4% (10)	0.021*
Metabolic syndrome	49.0% (51)	92.3% (12)	0.003*
SAH	22.1% (23)	42.9% (6)	0.105
Total cholesterol, mg/dL	186.0 ± 38.0	184.0 ± 37.0	0.739
LDL, mg/dL	110.0 ± 31.0	113.0 ± 32.0	0.874
HDL, mg/dL	46.0 ± 12.0	43.0 ± 9.0	0.524
Triglycerides, mg/dL	171.0 ± 107.0	173.0 ± 78.0	0.487
Smoking status			0.501
Current smoker	7.7% (8)	0.0%	
Former smoker	4.8% (5)	7.1% (0.8%-28.8%)	
Weight classification by BMI			0.912
Normal	3.8% (4)	-	
Overweight	22.1% (23)	14.3% (2)	

Obesity class I	30.8% (32)	28.6% (4)	
Obesity class II	27.9% (29)	35.7% (5)	
Obesity class III	15.4% (16)	21.4% (3)	
Waist circumference, cm	103.2 ± 14.5	112.4 ± 14.8	0.032*
WHR	0.9 ± 0.1	1.0 ± 0.0	0.012*

*p value < 0.05

NAFLD: nonalcoholic fatty liver disease; TE: transient elastography; SD: standard deviation; CAP: controlled attenuation parameter; NAFLD-FS: NAFLD fibrosis score; FIB-4: fibrosis-4; AST: aspartate aminotransferase; ALT: alanine aminotransferase; GGT: gamma glutamyl transferase; HA: hyperandrogenism and/or hyperandrogenemia; PCOS: polycystic ovary syndrome; DHEA-S: dehydroepiandrosterone sulfate; IR. insulin resistance; HOMA-IR. *Homeostasis Model Assessment of Insulin Resistance*; SAH. systemic arterial hypertension; LDL: low-density lipoprotein; HDL: high-density lipoprotein; BMI: body mass index; WHR: waist-hip ratio
